# Supplementary material for: An Evaluation of Oral Anticoagulant Safety Indicators by England’s Community Pharmacies
Source: Pharmacy (Basel). 2024 Aug 29;12(5):134. doi: 10.3390/pharmacy12050134 (PMC11417885; doi:10.3390/pharmacy12050134)
Supplement: Supplementary file 1 [file pharmacy-12-00134-s001.zip › pharmacy-3123043-supplementary.pdf]

PQS Oral Anticoagulant Safety Audit 2023/24 - Data Collection Form

Section 1 - All patients

1. Patient's name
(For internal use – not for reporting to NHS England)

2. Date / /

3. Patient's age

4. Is the patient a care home resident?
☐ Yes ☐ No ☐ Not known

5. Name of anticoagulant that the patient is taking*
☐ Acenocoumarol ☐ Phenindione
☐ Apixaban ☐ Rivaroxaban
☐ Dabigatran ☐ Warfarin
☐ Edoxaban

*for patients prescribed more than one anticoagulant, see question 7. This does not include where a patient is prescribed two strengths of the same medicines to make a dose e.g., multiple strengths of warfarin

6. Is the anticoagulant supplied in a monitored dosage system / compliance aid?
☐ No
☐ Yes, one medicine per blister / compartment
☐ Yes, multiple medicines per blister / compartment

7. Is the patient prescribed more than one oral anticoagulant? (Please do not include a patient prescribed two strengths of the same medicine to make a dose e.g., multiple strengths of warfarin)
☐ No (go to question 8)
☐ Yes

Name of other anticoagulant: ____________________

What action did you take and what was the outcome?

If patients are switching anticoagulant treatments, remind them to return any medicine no longer needed for safe disposal.

8a. Is the patient prescribed an oral NSAID* as well as the anticoagulant?
The PINCER summary[10] states that 'It is advisable to avoid this combination whenever possible'.
* **Do not** include low dose aspirin (300mg or less per day) here; record it in Q10 instead.

☐ No (go to question 9)
☐ Yes

8b. Is the patient also prescribed gastro-protection?
(e.g. a proton pump inhibitor or H2 receptor antagonist)
☐ Yes
☐ No

8c. Have you contacted the prescriber about concomitant use of an anticoagulant with an NSAID?
☐ Yes – prescriber discontinued anticoagulant and/or NSAID
☐ Yes – prescriber confirmed no medication changes required
☐ Yes – gastro-protection prescribed
☐ Yes – other action by prescriber. Please specify:
____

☐ No – please specify the reason:
____

9a. Is the patient prescribed an antiplatelet as well as the anticoagulant?

- ☐ No (go to question 10)
- ☐ Yes

9b.. Is the patient also prescribed gastro-protection?
(e.g. a proton pump inhibitor or H2 receptor antagonist)

The PINCER summary[10] indicates that gastro-protection should always be considered and offered when combination therapy (anticoagulant plus antiplatelet) is indicated.

- ☐ Yes
- ☐ No

9c. Have you contacted the prescriber for a review of gastro-protection?

- ☐ Yes – gastro-protection prescribed
- ☐ Yes – prescriber discontinued anticoagulant and / or antiplatelet
- ☐ Yes – prescriber confirmed no medication changes required
- ☐ No – prescriber has been contacted about gastro-protection for this patient within the last 6 months
- ☐ No – patient has discussed with prescriber and has made decision not to take gastro-protection
- ☐ Yes – other reason. Please specify:
- ☐ No – other reason. Please specify:

____

10. Which category best describes how the audit was completed for this patient?

- ☐ Conversation with the patient in the pharmacy
- ☐ Conversation with the patient by telephone
- ☐ Conversation with the patient by video link
- ☐ Contact with patient by other route, e.g. email

→ **Go to Section 2**

- ☐ Patient's representative in pharmacy, unable to contact patient
- ☐ Medicine delivered by pharmacy, unable to contact patient
- ☐ Care home patient, unable to contact patient / representative / care staff

→ **VKA prescribed – Go to Section 3**
DOAC prescribed – Go to Section 4

Section 2 - Patient feedback (only complete this section if you can contact the patient)

11. Was the patient already aware that they are taking an anticoagulant, i.e. a medicine to thin the blood/prevent blood clots?

- ☐ Yes
- ☐ No – information provided
- ☐ No – information not provided

12. Did the patient already know the symptoms of over-anticoagulation, e.g. unexplained bruising, nose bleeds?

- ☐ Yes
- ☐ No – information provided
- ☐ No – information not provided

13. Was the patient already aware of the need to check with the doctor or pharmacist before taking over-the-counter medicines, herbal products or supplements?

- ☐ Yes
- ☐ No – information provided
- ☐ No – information not provided

14. <u>For patients taking vitamin K antagonists only</u>

Was the patient already aware that dietary change can affect their anticoagulant medicine?

- ☐ Yes
- ☐ No – information provided
- ☐ No – information not provided
- ☐ Not applicable

15a. Did the patient have a standard yellow anticoagulant alert card?

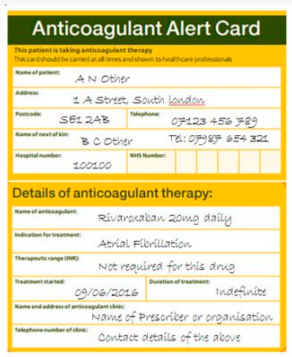

- ☐ Yes, card seen by pharmacy staff
- ☐ Yes, card not seen but patient confirmation they have this card
- ☐ No card but aware of card
- ☐ No card and unaware of card

15b. Was a standard yellow alert card offered to the patient?

- ☐ Yes, card accepted
- ☐ Yes, but card declined because the patient has manufacturer’s alert card
- ☐ Yes, but card declined because the patient has another anticoagulant alert card
- ☐ Yes, but card declined for other reason
- ☐ No, not offered. Reason - please specify

____

Vitamin K antagonist prescribed? Go to Section 3 **DOAC prescribed? Go to Section 4**

Section 3 - Patients prescribed vitamin K antagonists only

16a. Did you find out when the patient last had an INR test before issuing this medicine?

- ☐ No (go to question 16d)
- ☐ Yes

16b. How did you obtain this information? (select all that apply)

- ☐ From patient
- ☐ From patient's representative
- ☐ From yellow anticoagulant record book or other written record
- ☐ From general practice
- ☐ From patient's care provider, e.g. nursing home
- ☐ From anticoagulant service
- ☐ From other source - please specify:

____

16c. How long ago was the INR test?

- ☐ Fewer than 4 weeks (go to Section 4)
- ☐ 4 – 12 weeks (go to Section 4)
- ☐ More than 12 weeks

16d. If the INR test was more than 12 weeks ago, what, if any, action did you take?

(go to Section 4)

Section 4 – All patients

17. Please give details of any other referrals or action taken about anticoagulant safety issues, e.g. drug interactions, INR concern (do not include any patient identifiable information)
