# Supplementary figures and images for: An Evaluation of Oral Anticoagulant Safety Indicators by England’s Community Pharmacies
Source: Pharmacy (Basel). 2024 Aug 29;12(5):134. doi: 10.3390/pharmacy12050134 (PMC11417885; doi:10.3390/pharmacy12050134)

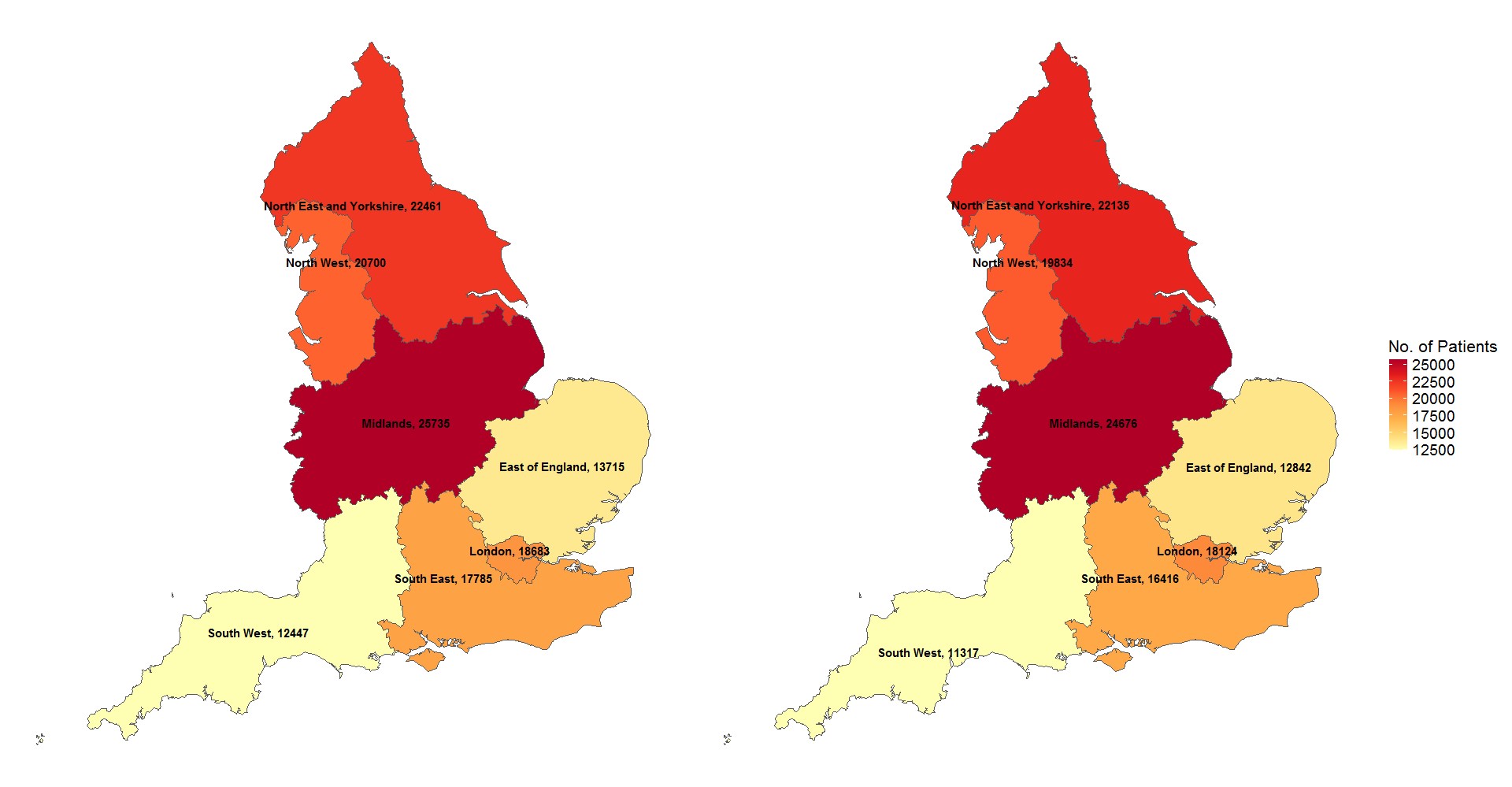

Supplement: Supplementary file 1 [file pharmacy-12-00134-s001.zip › map (1).jpg]
